# Three local plants adapt to ecological restoration of abandoned lead-zinc mines through assembly of rhizosphere bacterial communities

**DOI:** 10.3389/fmicb.2025.1533965

**Published:** 2025-02-10

**Authors:** Wei Gao, Shuyi Chen, Xin Yu, Sumin Chen, Caijing Wan, Ying Wang, Peng Wu, Qiang Li

**Affiliations:** ^1^Clinical Medical College & Affiliated Hospital of Chengdu University, Key Laboratory of Coarse Cereal Processing, Ministry of Agriculture and Rural Affairs, Chengdu, Sichuan, China; ^2^Yunnan Plateau Characteristic Agricultural Industry Research Institute, Yunnan Agricultural University, Kunming, Yunnan, China

**Keywords:** soil pollution, mining activities, bioremediation, microbial diversity, assembly mechanism

## Abstract

**Introduction:**

The plant restoration and ecological restoration of lead-zinc mines are very important.

**Methods:**

In this study, we used three local plants to carry out ecological restoration of abandoned lead–zinc mining areas and detected the adaptive mechanisms of soil bacterial diversity and function during the ecological restoration of lead–zinc mines through 16S rRNA sequencing.

**Results:**

The results revealed that lead-zinc mining significantly reduced the soil bacterial diversity, including the Shannon, Simpson, and observed species indices, whereas the planting of the three ecological restoration plants restored the soil microbial diversity to a certain extent, leading to increases in the Shannon index and Observed species indices. Mining activities significantly reduced the abundances of RB41 and *Bryobacter* in the bulk soil compared with those in the nonmining areas, whereas the three ecological restoration plants increased the abundances of RB41 and *Bryobacter* in the rhizosphere soil compared with those in the bulk soil in the mining areas. Following the planting of the three types of ecologically restored plants, the soil bacterial community structure partially recovered. In addition, different plants have been found to have different functions in the lead-zinc ecological restoration process, including iron complex transport system-permitting proteins and ATP binding cassettes.

**Discussion:**

This study confirms for the first time that plants adapt to the remediation process of abandoned lead-zinc mines by non-randomly assembling rhizosphere bacterial communities and functions, providing a reference for screening microbial remediation bacterial resources and plant microbe joint bioremediation strategies for lead-zinc mines.

## Introduction

Lead-zinc ore, a crucial nonferrous metal mineral, is highly important in industry worldwide. Lead–zinc alloys and their derivatives find extensive applications in sectors such as automobiles, batteries, construction, and chemicals, thereby playing a pivotal role in fostering global economic growth (Li D. et al., 2024). However, the mining and smelting of lead-zinc ores have led to substantial environmental challenges (Chen et al., 2023; Chen T. et al., 2022). During these processes, considerable amounts of dust and exhaust gases, primarily composed of sulfides and nitrogen oxides, are emitted (Garry et al., 2018; Kan et al., 2021). These pollutants not only severely impact the air quality surrounding mining areas but also pose a potential threat to the global atmospheric environment through atmospheric dissemination (Peng et al., 2023). Furthermore, mining and smelting generate wastewater laden with heavy metal ions and acidic substances, particularly lead, zinc, and cadmium (Guo et al., 2023; Ma et al., 2023). Direct discharge of this untreated wastewater into water bodies such as rivers and lakes poses risks to aquatic life and threatens human health through the food chain (Wang et al., 2022; Wang et al., 2023). Solid waste, including waste rocks and residues from mining, can also contaminate soil (Junusbekov et al., 2023; Sun et al., 2022). Prolonged accumulation can degrade soil structure and fertility, adversely affecting crop growth and yield (Qiao et al., 2022). Studies have indicated that lead–zinc mining can systematically affect the surrounding ecosystem, resulting in a decline in biodiversity (Kastury et al., 2023; Larsen et al., 2001). Individuals exposed to the mining environment for extended periods are susceptible to heavy metal accumulation and irreversible bodily tissue damage (Choudhari et al., 2010). Therefore, it is imperative to undertake ecological restoration in lead–zinc mining areas and mitigate their ecological risk (Paluchamy and Mishra, 2022; Yang et al., 2023).

The approaches for the ecological remediation of lead-zinc mine pollution include physical, chemical, and biological methods (Cai et al., 2021; Sha et al., 2023). Physical methods involve precipitation, filtration, and adsorption, whereas chemical methods include neutralization, oxidation–reduction, and precipitation, all aimed at reducing lead and zinc concentrations in soil and water (Jiang S. et al., 2022; Lei et al., 2018; Luo et al., 2022). However, these methods are costly and prone to secondary pollution. Biological methods that leverage the remediation capabilities of plants and microorganisms are cost-effective and environmentally friendly (Su et al., 2023; Diallo et al., 2024; Duan et al., 2022). Phytoremediation, an *in situ* technique, avoids secondary ecosystem pollution (Su et al., 2022). By cultivating locally adapted plants, vegetation cover in mining areas can be restored, increasing soil quality (Li et al., 2018). Recently, plant remediation technology has garnered extensive attention and research, with ongoing efforts to screen and cultivate plants with superior remediation capabilities to increase efficiency (Tang et al., 2019; Xiao et al., 2022). Additionally, the integration of plant restoration with other techniques, such as plant–microbe combined restoration, has emerged as a research focus (Xiao et al., 2022; Han et al., 2020). Through symbiotic relationships, plants and microorganisms efficiently remove pollutants, utilizing the absorption and transformation abilities of plants and the degradation and transformation capabilities of microorganisms (Chen J. et al., 2022; Jiang X. et al., 2022; Singh et al., 2022). Microorganisms can adsorb harmful substances, removing them from the environment by binding to pollutants through surface viscous substances or extracellular polymeric substances (Li et al., 2022a; Li Q. et al., 2024). They can also metabolically reduce heavy metal pollutants, converting them into harmless forms (Dang et al., 2021; Bao et al., 2023). Moreover, microorganisms secrete growth-promoting substances, regulate plant tolerance and adsorption capacity for heavy metals, and improve soil conditions through long-term coevolution and mutual adaptation with plants (Jacob et al., 2018; Noor et al., 2022; Ojuederie and Babalola, 2017).

Currently, research on *in situ* phytoremediation of lead–zinc mines is limited, and plant adaptability and remediation capabilities vary across environments. The composition and role of different plant rhizosphere bacterial communities in phytoremediation processes remain unclear (Tang et al., 2022). In this study, we cultivated three lead–zinc accumulator plants—*Carex nubigena*, *Pteris cretica* L. var. *nervosa*, and *Neyraudia reynaudiana*—in a lead–zinc mining area and adjacent nonmining areas. These plants are known for their ability to accumulate lead and zinc, strong stress resistance, and local adaptability (Li et al., 2018; He et al., 2022; He et al., 2023; Liu et al., 2020). Five years post-planting, the ecological restoration plants demonstrated robust growth. To understand the basis of their efficient environmental adaptability and remediation capabilities, we analyzed changes in the composition and diversity of rhizosphere bacterial communities in mining and nonmining areas via 16S rRNA high-throughput sequencing. Our findings contribute to the screening of plant growth-promoting bacteria and bioremediation strains, providing valuable insights for ecological restoration in lead–zinc mining areas.

## Materials and methods

### Cultivation and management strategies for ecologically restored plants

The Ya’an lead–zinc mine, an abandoned mining site, necessitates robust ecological restoration efforts to mitigate the risks associated with pollutant exposure and dissemination. In 2019, we embarked on an initiative to ecologically restore this mining area by selecting three lead/zinc-tolerant plants: *Carex nubigena*, *Pteris cretica* L. var. *nervosa*, and *Neyraudia reynaudiana*. These plants were strategically planted every meter within the mining area and adjacent nonmining areas to ensure uniform distribution and balanced data collection. Postplanting, minimal watering was provided—only 2–3 times within the first month—to facilitate plant establishment without relying on exogenous inputs such as fertilizers (Deng et al., 2024). Five years later, rhizosphere soils from both mining and nonmining areas were collected for bacterial diversity sequencing.

### Rhizosphere soil collection and DNA extraction

Rhizosphere soil samples were meticulously collected from *Carex nubigena*, *Pteris cretica* L. var. *nervosa*, and *Neyraudia reynaudiana* in both lead-zinc mining-affected and nonmining regions. Plants exhibiting consistent growth patterns were chosen, and the soil adhering to their roots was gently shaken into self-sealing bags. Approximately 200 grams of soil per plant were collected for bacterial diversity sequencing. Additionally, bulk soil samples from both mining and nonmining sites served as comparators, with each sample comprising three biological replicates. The rhizosphere soils from the mining area were designated Mine. C (*Carex nubigena*), Mine. P (*Pteris cretica* L. var. *nervosa*), Mine. N (*Neyraudia reynaudiana*) and Mine (bulk soil), while those from nonmining areas were designated the control. C, Control. P, Control. N, and Control, respectively. Each soil sample was replicated three times (*n* = 3), with 50 grams of each sample allocated for bacterial diversity analysis and isolation. The 24 samples were transported to the laboratory in ice bags for DNA extraction and 16S rRNA sequencing. Genomic DNA was extracted via an Omega soil DNA kit (D5625-02, CA, United States), and the quality of the extracted DNA was verified via 1% agarose gels.

### PCR amplification and detection

The extracted genomic DNA was diluted to a concentration of 1 ng/μL in sterile water and then amplified via primers targeting the 16S rRNA V3–V4 regions. These primers incorporated a unique barcode for sample identification. The PCR mixture contained 15 μL of Phusion^®^ High-Fidelity PCR Master Mix (New England Biolabs), 2 μM forward and reverse primers, and 10 ng of template DNA. The PCR protocol involved an initial denaturation step at 98°C for 1 min, followed by 30 cycles of denaturation at 98°C for 10 s, annealing at 50°C for 30 s, extension at 72°C for 30 s, and a final extension at 72°C for 5 min (Gao et al., 2024). The PCR products were mixed with an equal volume of SYBR green-containing loading buffer and subjected to electrophoresis on a 2% (w/v) agarose gel for detection. Purified PCR products were obtained via the Qiagen Gel Extraction Kit (Qiagen, Germany).

### Library preparation, sequencing, and data processing

The sequencing libraries were constructed following the manufacturer’s instructions via the TruSeq^®^ DNA PCR-Free Sample Preparation Kit (Illumina, United States), with index codes included for sample identification. The quality of the libraries was assessed via a Qubit@ 2.0 fluorometer (Thermo Scientific, United States) and an Agilent Bioanalyzer 2,100 system. Sequencing was performed on the Illumina NovaSeq platform, generating 250-bp paired-end reads. Reads were identified by their unique barcodes and processed to remove barcode and primer sequences. FLASH V1.2.7 (Magoc and Salzberg, 2011) was used to merge paired-end reads, and raw tags were filtered on the basis of quality control criteria via QIIME V2 (Hall and Beiko, 2018). Chimeric sequences were identified by comparing tags with the reference Silva database and removed from the dataset (Quast et al., 2013).

### Operational taxonomic units clustering and species annotation

Sequences with a similarity of ≥97% were grouped into operational taxonomic units (OTUs) via Uparse v7.0.1001 (Edgar, 2013). A representative sequence was selected for each OTU for annotation. Taxonomic information was assigned to each representative sequence via the Silva database in conjunction with the Mothur algorithm (Quast et al., 2013). Multiple sequence alignments were performed via MUSCLE v3.8.3 to investigate the phylogenetic relationships between OTUs and changes in the most abundant species across different samples or groups (Edgar, 2004). To standardize the OTU abundance information, a sequence number benchmark was applied on the basis of the sample with the smallest number of sequences. The normalized data were used for further analysis of alpha and beta diversity.

### Alpha and beta diversity analyses

To assess species diversity complexity within specific samples, six indices—observed species, Chao1, Shannon, Simpson, ACE, and Good’s coverage—were considered. These indices were calculated via QIIME v2 and presented via R v2.15.3 (Hall and Beiko, 2018). Two indices—observed species and the Chao1 estimator—were chosen to measure community richness. The Shannon and Simpson indices were used to quantify community diversity. Beta diversity analysis was conducted to evaluate differences in species complexity between samples. Nonmetric multidimensional scaling (NMDS) and principal coordinate analysis (PCoA) were performed via the R vegan software package.

### Functional prediction of rhizosphere bacteria

Functional predictions of rhizosphere bacteria were made via Tax4Fun (Aßhauer et al., 2015). Projections were based on the KO database to gain insights into the potential functions of these bacteria.

### Statistical analysis

Statistical analysis was conducted to evaluate differences between samples. The *t* test was used for comparing two sets of samples, whereas Tukey’s test was applied for comparisons involving more than two samples (Qiu et al., 2024). A *p* value <0.05 was considered indicative of statistically significant differences among different groups. Benjamini Hochberg adjustment is used for multiple comparison correction in data analysis.

## Results

### Sequencing data analysis

**Figure 1:**
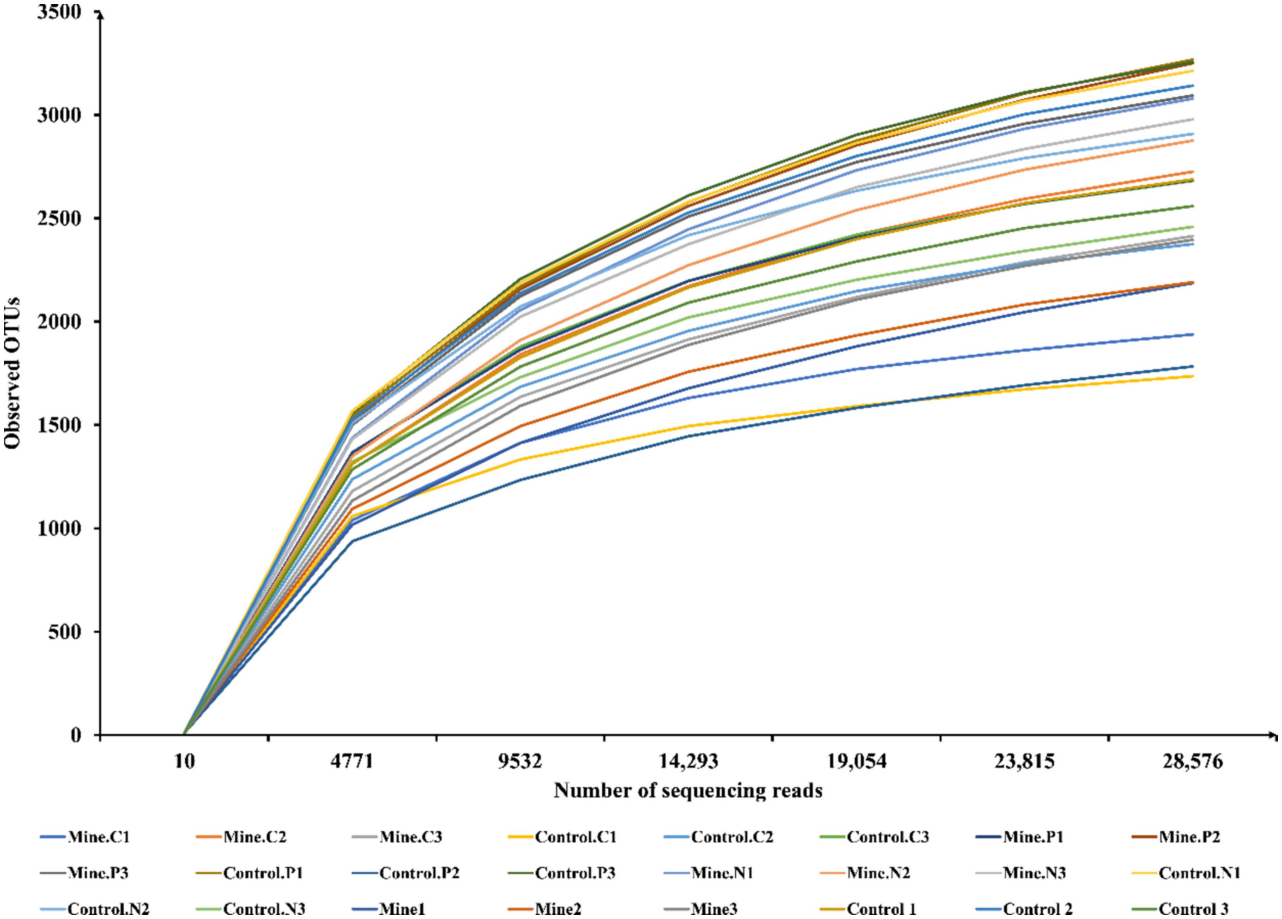
Trends of observed OTUs in different samples changing with sequencing reads.

In our investigation, we investigated the assembly mechanism of rhizosphere bacterial communities linked to *Carex nubigena*, *Pteris cretica* L. var. *nervosa*, and *Neyraudia reynaudiana* during the ecological restoration process in lead–zinc mining areas. Across all the samples, we obtained an average of 63,855 raw reads per sample (Supplementary Table S1). Subsequent filtering of chimeras, low-quality sequences, and short sequences yielded an average of 47,824 effective reads per sample for in-depth analysis. Using rarefaction curves (Figure 1), we evaluated whether the observed OTUs varied with the number of sequencing reads. As the sequencing depth increased, the number of observed OTUs progressively increased. Notably, once the sequencing read count surpassed 23,815, the rarefaction curves flattened out, indicating that our sequencing depth was sufficient to capture the comprehensive community structure and diversity of rhizosphere bacteria. We classified these reads into OTUs using a 97% similarity threshold, ultimately identifying a total of 4,447 OTUs across all eight samples (with each sample having three biological replicates).

### Alpha diversity indices

**Figure 2:**
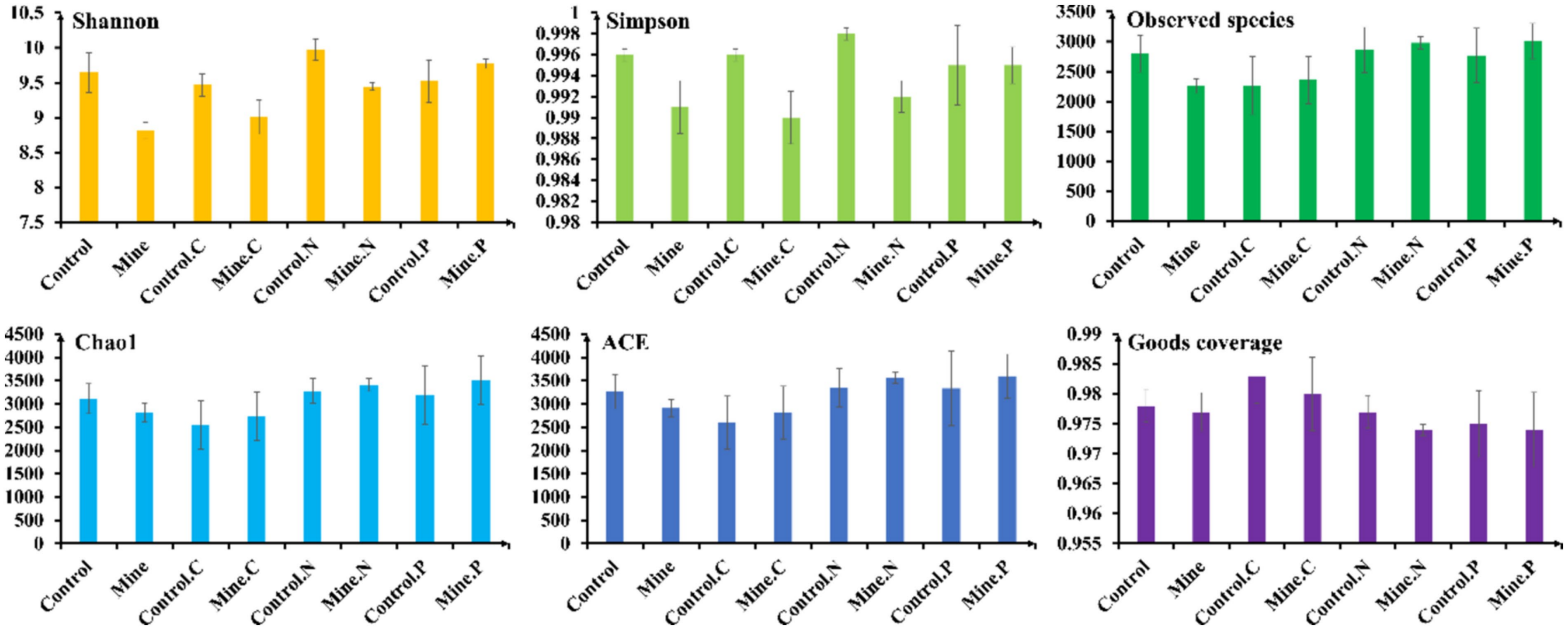
Changes in the alpha diversity indicators of different samples (*n* = 3).

Compared with bulk soils from nonmining areas, lead-zinc mining significantly reduced the bacterial diversity index and community richness of bulk soils in mining areas, as evidenced by the Shannon, Simpson, and observed species indices (Figure 2). These findings underscore the substantial impact of lead-zinc mining on the bacterial diversity and community composition of mining area soils. However, there were no notable differences in the Chao1 and ACE indices between bulk soils from mining areas and those from nonmining areas. Intriguingly, the planting of the three plant species in mining area soils somewhat mitigated this decline, increasing the diversity and community richness of rhizosphere bacteria. Specifically, *Neyraudia reynaudiana* and *Pteris cretica* presented significantly greater Shannon indices and observed species indices in mining area soils than in bulk soil from the same area. Furthermore, the Chao1 and ACE indices of *Neyraudia reynaudiana* were also significantly greater in mining area soils than in bulk soils. Compared with those of control plants from nonmining areas, the Shannon and Simpson indices of *Carex nubigena* and *Neyraudia reynaudiana* from mining areas were notably lower, whereas no significant changes in other plants or alpha diversity indices were detected.

### Taxonomic analyses of bacterial communities

**Figure 3:**
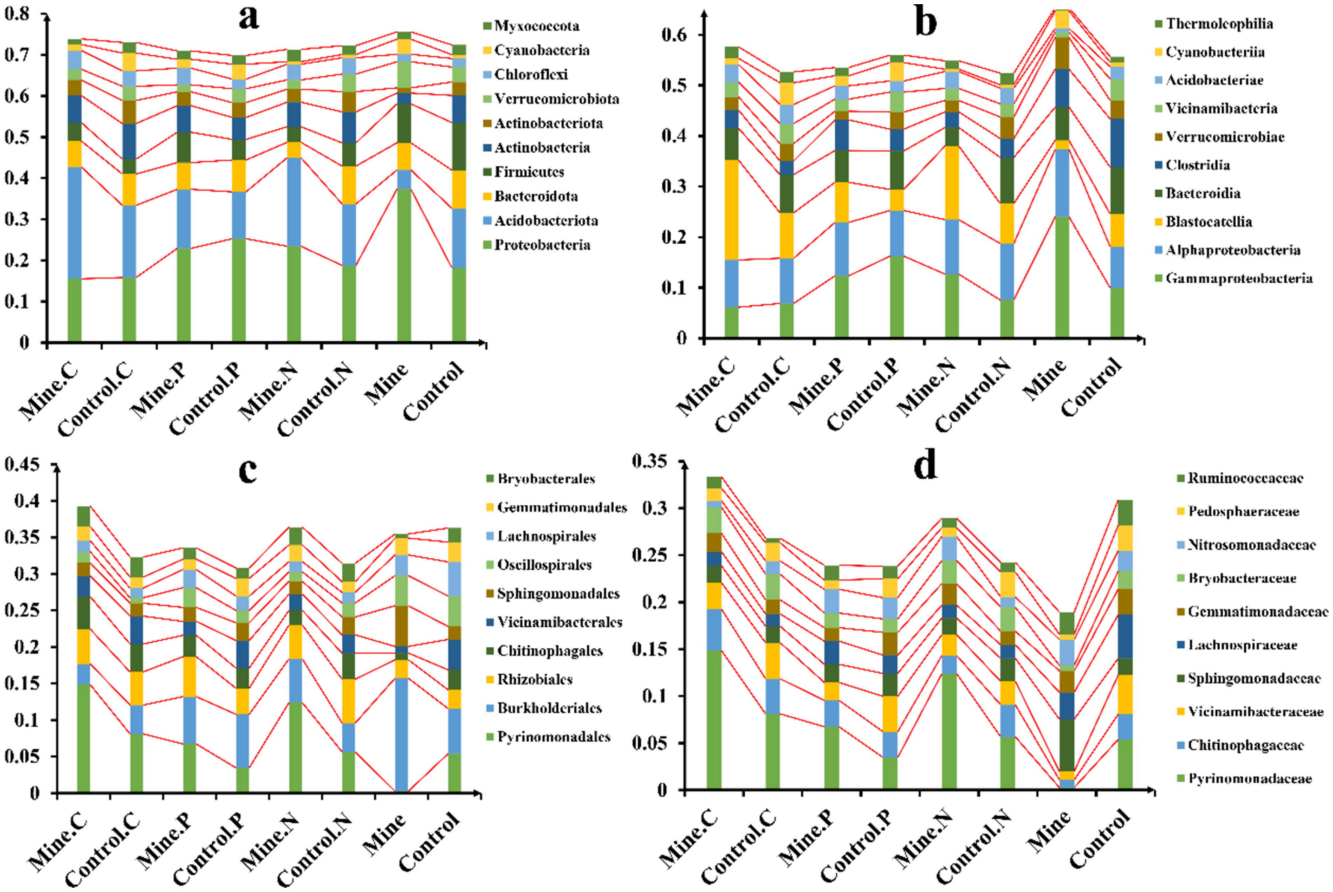
Relative abundance of soil bacteria in different samples at the phylum **(A)**, class **(B)**, order **(C)**, and family **(D)** levels.

In this study, a comprehensive taxonomic analysis of bacterial communities was conducted across various samples. A total of 101 bacterial phyla were identified, with the abundances of the top 10 most prevalent phyla being compared (Figure 3A). Notably, Proteobacteria was the dominant phylum in all the samples, accounting for 22.15% of the total bacterial population. This was followed by Acidobacteriota (average 15.78%), Bacteroidota (average 7.12%), and Firmicutes (average 6.38%). Mining activities had a significant effect on the bacterial community structure. Specifically, they led to an increase in the abundance of soil Proteobacteria and a decrease in Acidobacteriota. However, the introduction of three ecological restoration plants in the mining area reversed these trends. Compared with that in the bulk soil from the mining area, the abundance of Proteobacteria significantly decreased, whereas the abundance of Acidobacteriota significantly increased in the rhizosphere soil of these plants. Among the different plants, *Carex nubigena* and *Neyraudia reynaudiana* from the mining area presented a significant increase in Acidobacteriota abundance compared with the rhizosphere soil of control plants from nonmining areas.

At the class level, a total of 203 bacterial classes were detected (Figure 3B). Gammaproteobacteria was the most abundant class, averaging 11.98% across all the samples, followed by Alphaproteobacteria (average 10.16%), Blastocatellia (average 8.94%), and Bacteroidia (average 7.04%). Compared with those in nonmining areas, mining activities increased the abundance of Gammaproteobacteria and Alphaproteobacteria in bulk soil while decreasing the abundance of Blastocatellia. Compared with those in the bulk soil from the mining area, the abundances of Gammaproteobacteria and Alphaproteobacteria in the rhizosphere soil and the abundance of Blastocatellia were lower in the ecological restoration plants. Furthermore, the rhizosphere soil of the three plant species from the mining area presented an increased abundance of Blastocatellia compared with the rhizosphere soil of the same plant species from nonmining areas.

Among the orders (Figure 3C), Pyrinomonadales (7.10%), Burkholderiales (6.52%), Rhizobiales (4.26%), and Chitinophagales (2.90%) were the most abundant. Mining activities reduced the abundance of Pyrinomonadales and Chitinophagales while increasing Burkholderiales in the bulk soil. However, the rhizosphere soil of the three ecologically restored plants presented an increased abundance of Pyrinomonadales and a decreased abundance of Burkholderiales compared with those in the bulk soil from the mining area. Additionally, the rhizosphere soil of the three plant species from the mining area had a greater abundance of Pyrinomonadales than did the rhizosphere soil of the corresponding control plants from nonmining areas.

At the family level (Figure 3D), Pyrinomonadaceae was the most abundant family, followed by Chitinophagaceae, Vicinamibacteraceae, and Sphingomonadaceae. Compared with those in nonmining areas, the abundances of Pyrinomonadaceae, Chitinophagaceae, and Vicinamibacteraceae in bulk soils from mining areas decreased. However, the planting of ecologically restored plants significantly increased their abundance in mining area soils. Specifically, the rhizosphere soil of the three plant species from the mining area presented a significant increase in Pyrinomonadaceae abundance compared with that of the control samples from nonmining areas.

**Figure 4:**
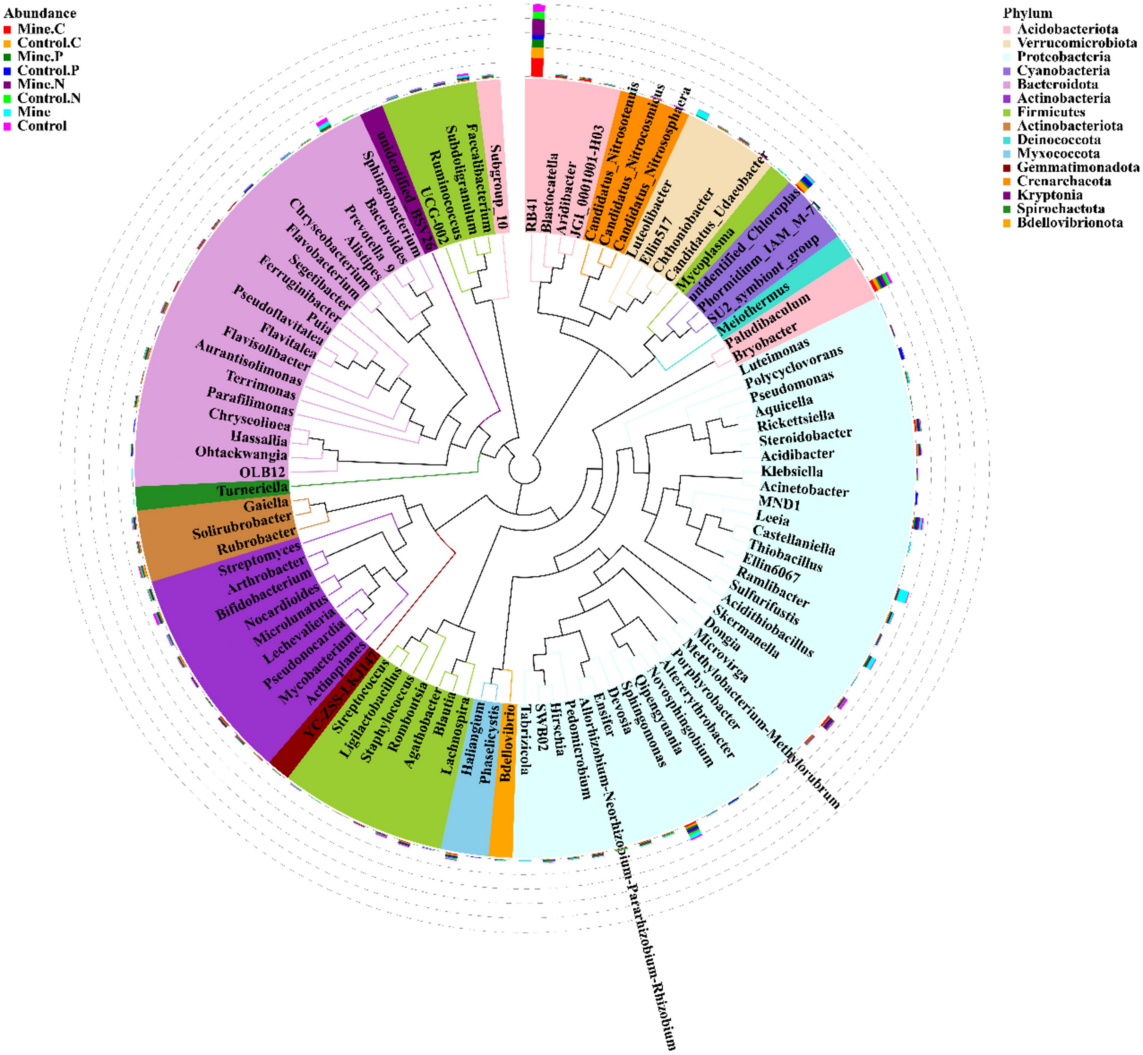
Phylogenetic analysis of the top 100 genera with relative abundances in different samples.

**Figure 5:**
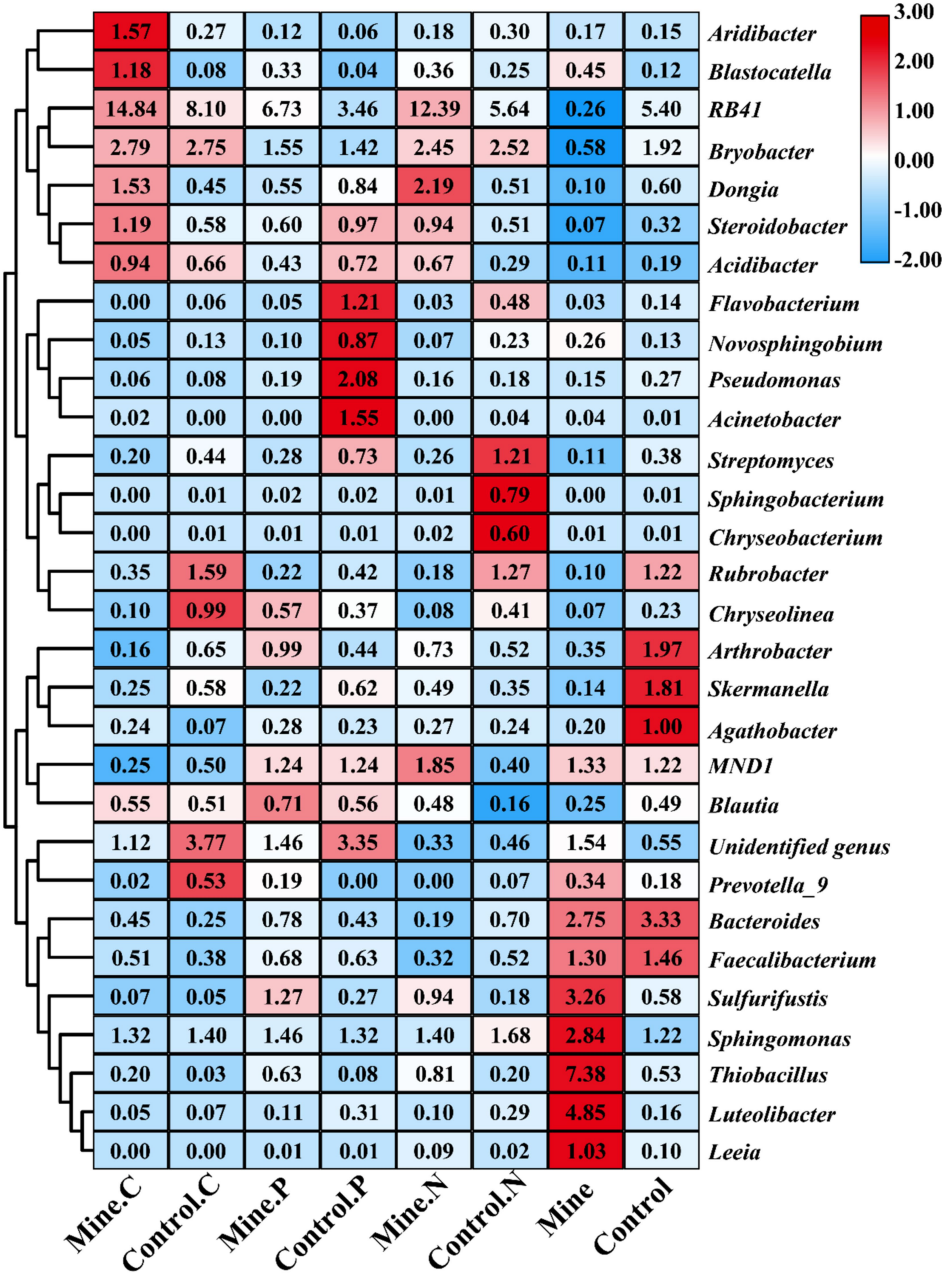
Cluster heatmap of the relative abundance of soil bacteria at the genus level for different samples. Different color blocks represent changes in the relative abundance of bacteria, with higher relative abundance resulting in darker colors. The numbers in the color block represent the relative abundance (%) of bacterial genera.

At the genus level, RB41 was the most abundant genus, followed by *Bryobacter*, *Sphingomonas*, *Thiobacillus*, and *Bacteroides* (Figure 4). Compared with those in nonmining areas, mining activities significantly decreased the abundances of RB41 and *Bryobacter* in bulk soils while increasing the abundances of *Sphingomonas* and *Thiobacillus* (Figure 5). Compared with those in the bulk soil, the abundances of RB41 and *Bryobacter* in the rhizosphere soil increased in the mining area. Conversely, the abundances of *Sphingomonas*, *Thiobacillus*, and *Bacteroides* significantly decreased in the rhizosphere soil of the mining area. The abundances of RB41 and *Thiobacillus* in the three plant species from the mining area increased compared with those in the control rhizosphere soil samples from nonmining areas, whereas the abundance of *Luteolibacter* decreased. Additionally, different plants enriched different bacterial groups in the rhizosphere soil of mining areas compared with nonmining areas. For example, *Blastocatella*, *Aridibacter*, and *Puia* were significantly enriched in the rhizosphere soil of *Carex nubigena* plants from mining areas, whereas *Pseudonocardia* and *Aquicella* were enriched in the rhizosphere soil of *Pteris cretica* plants. *Dongia* and *Pseudonocardia* were enriched in the rhizosphere soil of *Neyraudia reynaudiana* plants from mining areas.

### Structural distinctiveness of microbial communities

**Figure 6:**
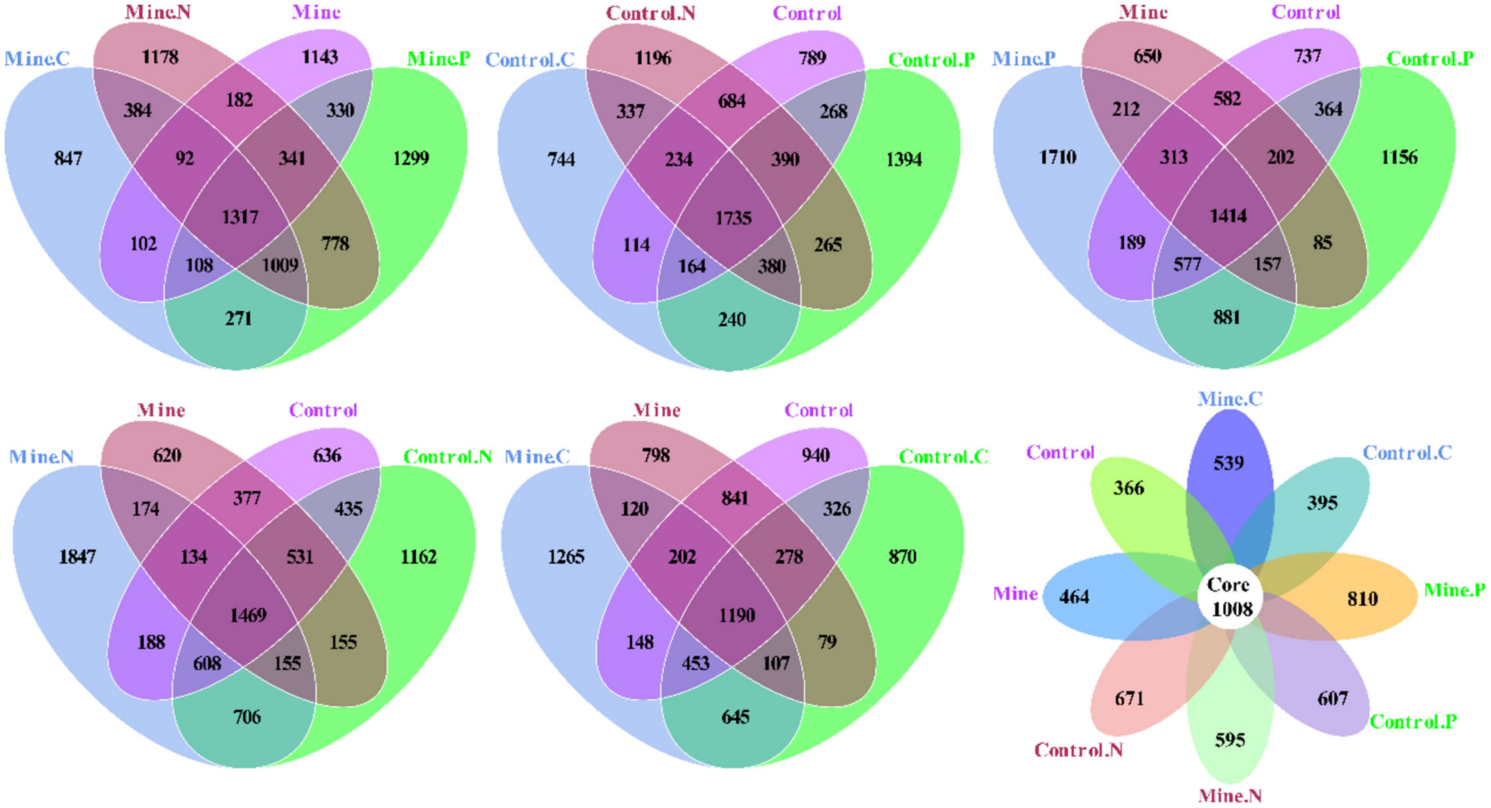
Shared and unique OTU analysis among different samples.

In this study, we conducted an in-depth analysis of the specific and overlapping operational taxonomic units (OTUs) among diverse samples (Figure 6). Specifically, the mining area samples—Mine. C, Mine. N, Mine. P, and Mine collectively contained 847, 1,178, 1,299, and 1,143 unique OTUs, respectively, with a shared pool of 1,317 OTUs. Conversely, the nonmining area samples—control. C, Control. N, Control. P, and Control—featured 744, 1,196, 1,394, and 789 unique OTUs, respectively, and a shared complement of 1735 OTUs. Furthermore, Mine. C and Control. C harbored 1735 and 1,553 unique OTUs, respectively, with 2,395 common OTUs. Similarly, Mine. N and Control. N presented 2,343 and 2,283 unique OTUs, respectively, sharing 2,938 OTUs. Mine. P and Control. P had 2,424 and 1807 unique OTUs, respectively, with 3,029 common OTUs. Notably, compared with nonmining areas, lead-zinc mining introduced 1,104 unique OTUs and retained 2,511 common OTUs in the bulk soil. Across all the samples, a range of 366–810 unique OTUs and 1,008 shared OTUs was observed.

**Figure 7:**
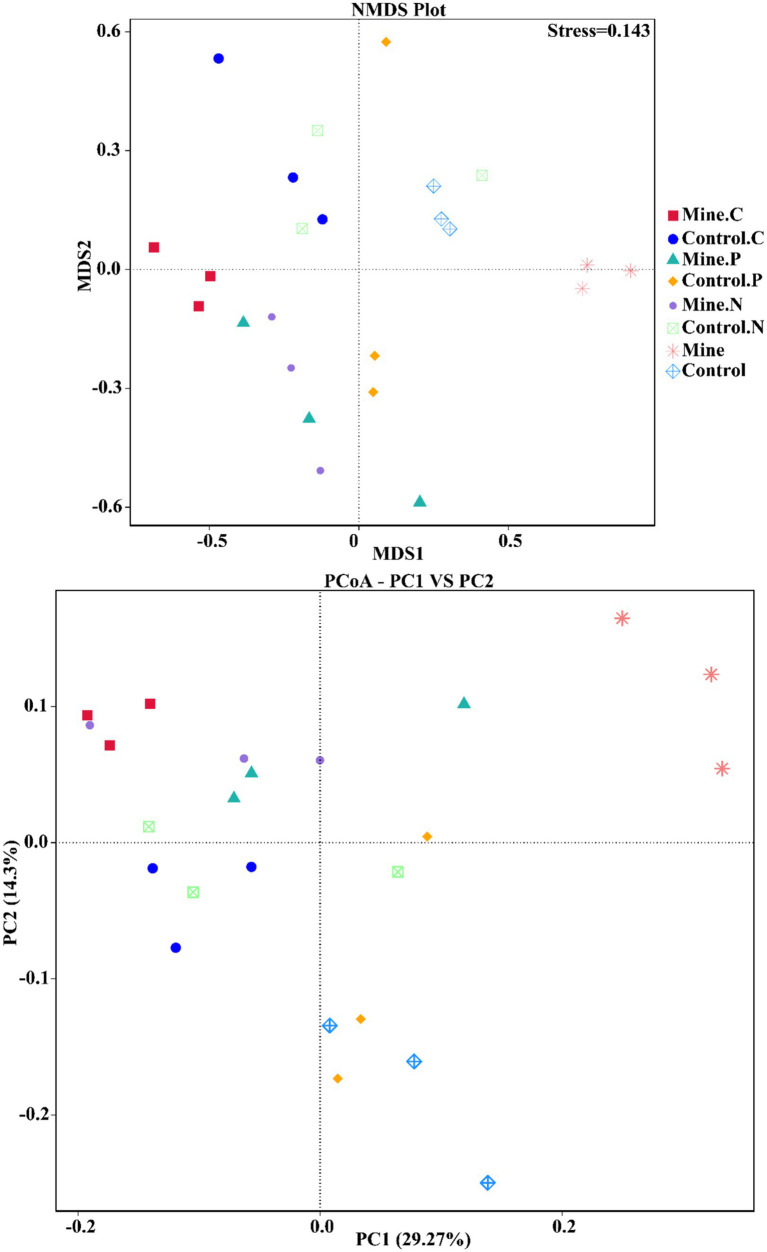
Beta diversity between different samples based on NMDS and PCoA.

To quantify the variations in bacterial communities among the samples, we employed principal coordinate analysis (PCoA) and nonmetric multidimensional scaling (NMDS) (Figure 7). The results revealed a striking difference in the bacterial community structure of the bulk soil samples from the mining area compared with that of the other samples, confirming that lead-zinc mining substantially altered the bacterial community structure of the soil. Following the planting of the three types of ecological restoration plants, the soil bacterial community structure partially recovered, resembling the community structure of the control sample. Moreover, the rhizosphere bacterial community structure of different plants within mining areas exhibited notable differences.

### Functional prediction of bacterial communities

**Figure 8:**
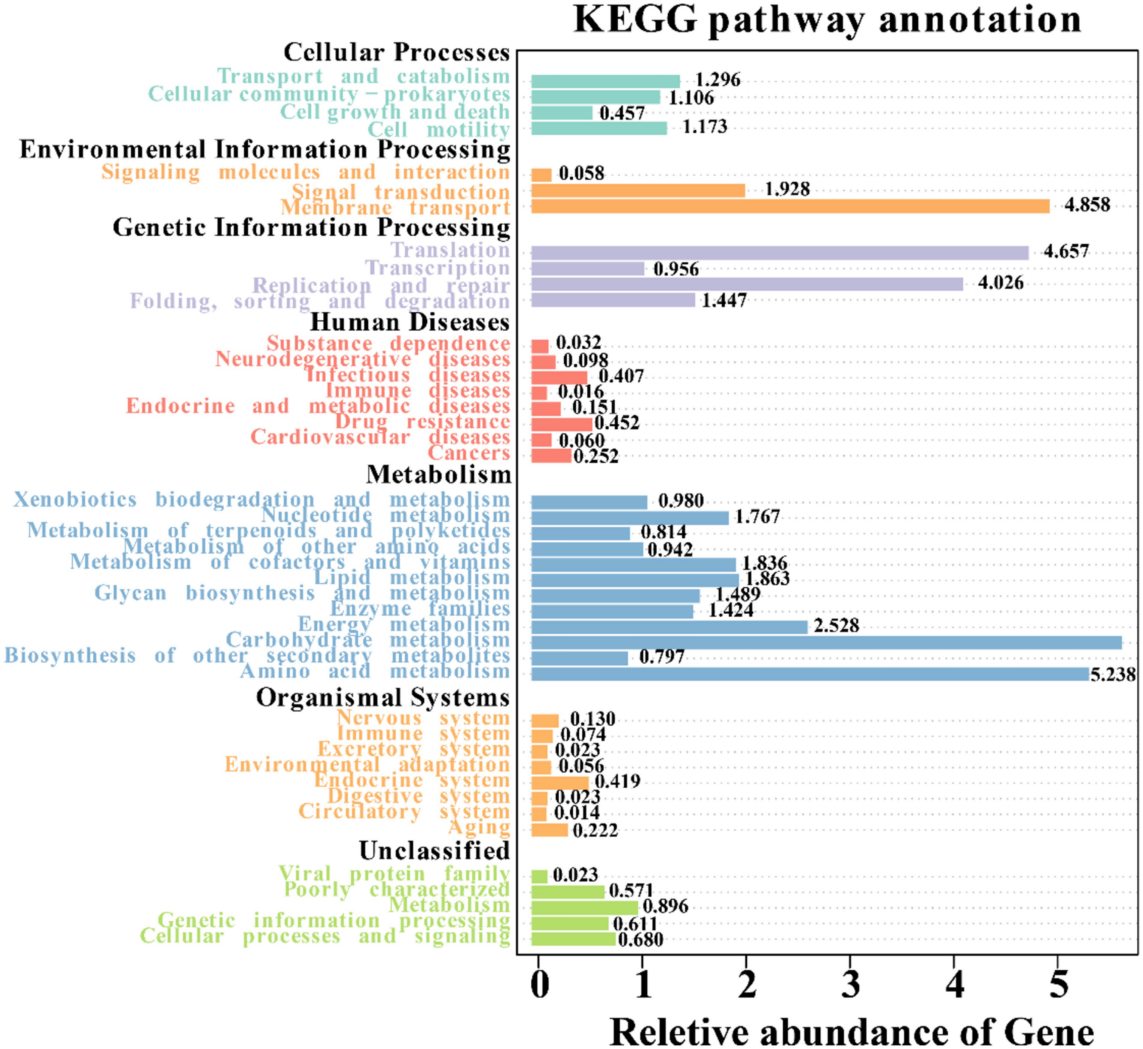
Annotation of KEGG pathways of soil bacteria in different samples based on Tax4Fun prediction.

By utilizing Tax4Fun, we forecasted the bacterial functions within the soil samples. These functions were categorized into 44 groups, with carbohydrate metabolism topping the list, followed by amino acid metabolism, membrane transport, translation, replication and repair, and energy metabolism (Figure 8). When comparing bulk soil from mining and nonmining areas, mining area soil was enriched in functions such as methyl-accepting chemical protein (K03406), Cu^2+^-exporting ATPase (K01533), and DNA-directed RNA polymerase subunit beta (K03046). Conversely, functions such as methyl-accepting chemotaxis protein excinuclease ABC subunit A (K03701), 3-oxoacyl-[acyl-carrier protein] reductase (K00059), and ribonuclease E (K08300) were significantly depleted.

**Figure 9:**
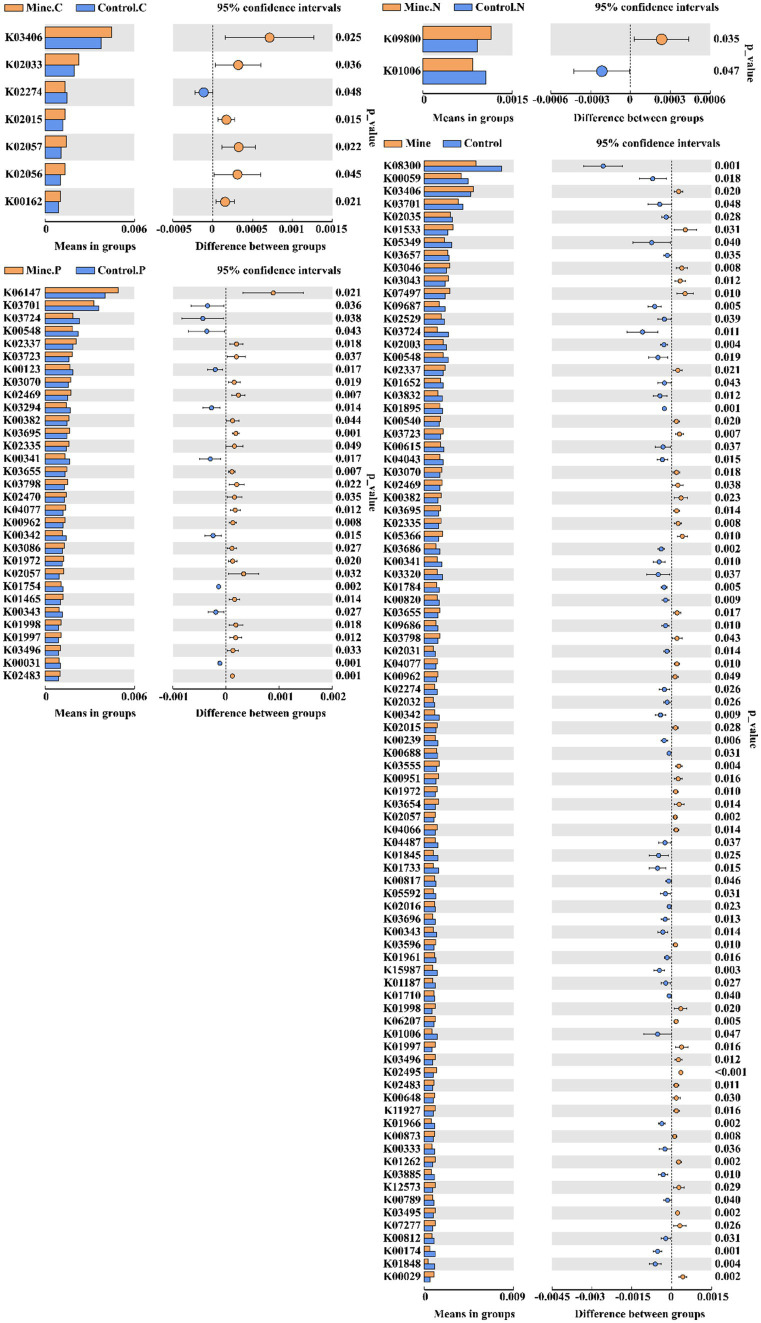
Comparative analysis of KO functions with significant differences between different samples.

In the rhizosphere soil of plants from mining areas, *Carex nubigena* increased functions related to iron complex transport system permease protein (K02015), peptide/nickel transport system permease protein (K02033), and methyl-accepting chemotaxis protein (K03406) but decreased the function of cytochrome c oxidase subunit I (K02274) (Figure 9). *Neyraudia reynaudiana* plants from mining areas enriched the function of a hypothetical protein (K09800) but decreased the functions of pyruvate and orthophosphate dikinase (K01006) compared with those in control rhizosphere soil samples from nonmining areas. Additionally, in the rhizosphere soil of *Pteris cretica* from mining areas, the levels of transcription-repair coupling factor (K03723), DNA polymerase III subunit alpha (K02337), and ATP-binding cassette (K06147) genes increased significantly, whereas the levels of 5-methyltetrahydrofolate–homocysteine methyltransferase (K00548), ATP-dependent helicase Lhr and Lhr-like helicase (K03724), and excinuclease ABC subunit A (K03701) genes decreased compared with those in nonmining area samples.

**Figure 10:**
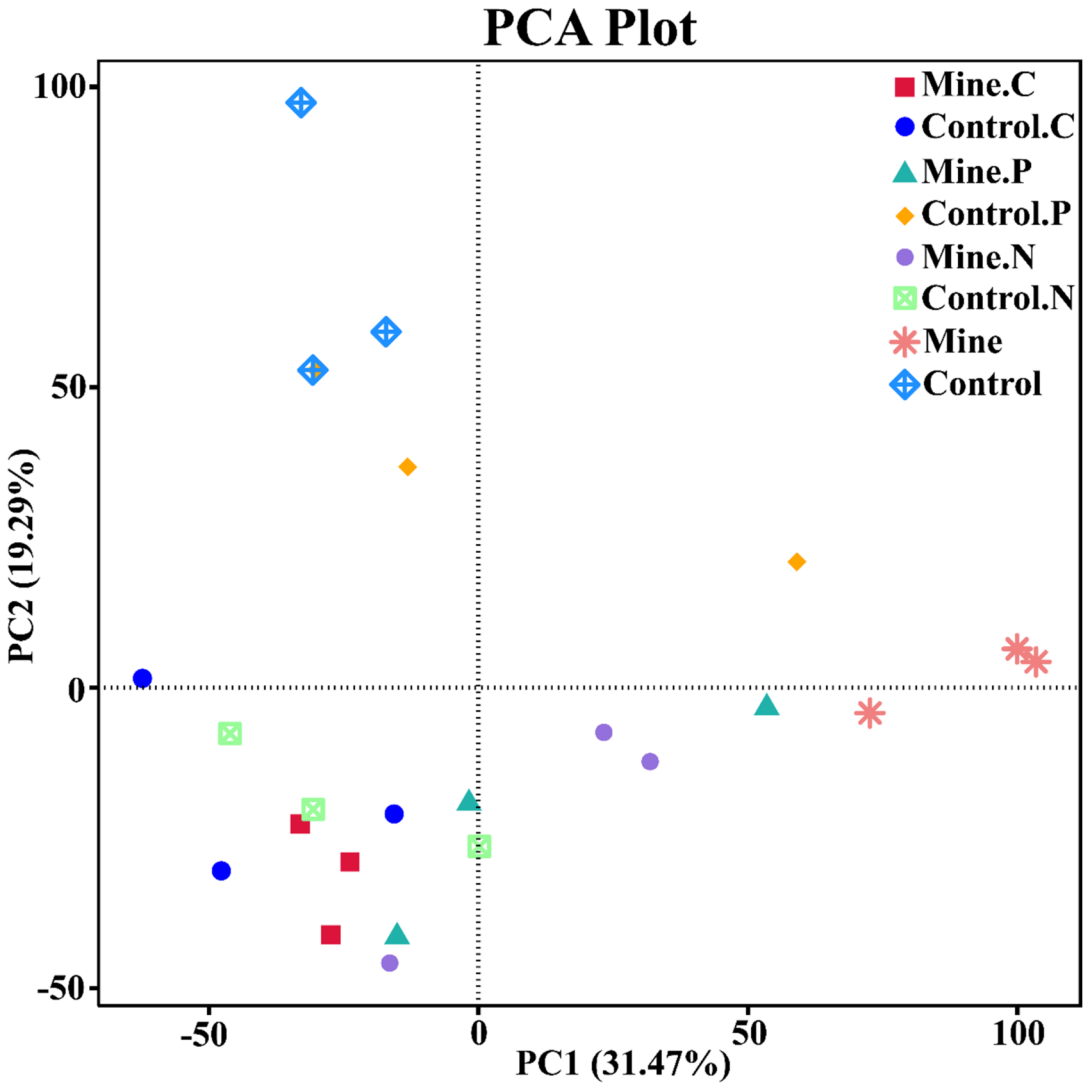
PCA of soil bacterial functions in different samples based on Tax4Fun prediction.

To further assess the variations in bacterial functions across samples, we applied principal component analysis (PCA) (Figure 10). Compared with the control samples, the lead–zinc mining samples induced significant differences in soil bacterial functions. The introduction of ecologically restored plants somewhat restored the soil bacterial functions, making them closer to those of the control samples. Notably, the functions associated with different plants underwent a certain degree of differentiation.

## Discussion

Abandoned lead–zinc mines can generate waste gas, wastewater, and solid waste (Cao et al., 2023; Fernández-Martínez et al., 2024). If left untreated, it may lead to the spread of ecological risks, further causing harmful elements such as heavy metals to harm human and other animal and plant health through the food chain (Xu et al., 2024; Yohannes et al., 2022). The characteristics of plant ecological restoration include low energy consumption, low cost, and environmental friendliness (Hassan et al., 2024; Narayanan et al., 2021; Thomas et al., 2022). Plants can fix or adsorb soil pollutants on their own and cooperate with rhizosphere bacteria, preventing soil erosion and pollutant diffusion in abandoned mining areas (Doku et al., 2024; Duan et al., 2021; Xiong et al., 2024). In the process of plant ecological restoration, rhizosphere bacteria play crucial roles, including assisting plants in fixing and adsorbing pollutants, enhancing plant stress resistance and environmental adaptability, and promoting plant growth (Xiao et al., 2022; Bennis et al., 2022; Xiao et al., 2024). In this study, three local plants were selected for the ecological restoration of abandoned lead–zinc mining areas. The results revealed that lead–zinc mining significantly reduced the soil bacterial diversity, whereas after years of plant planting, the soil bacterial diversity was restored to a certain extent, which is consistent with previous research findings (Deng et al., 2024). Research has shown that microbial diversity is closely related to the health level of plants (Berg et al., 2017; Trivedi et al., 2020). The greater the microbial diversity is, the greater the ability of the plant to adapt to the environment (Banerjee and van der Heijden, 2023; Cheng et al., 2019). The rhizosphere bacterial diversity of *Neyraudia reynaudiana* and *Pteris cretica* plants was most significantly restored in the soil of abandoned mining areas, indicating that these two plants can adapt well to the environment of lead–zinc mining and respond to environmental stress by increasing rhizosphere bacterial diversity. In the subsequent ecological restoration process, the planting of these two plants can be increased to improve their role in ecological restoration (Li et al., 2018; He et al., 2022).

This study revealed that mining activities significantly altered the soil community composition, reducing the abundance of soil RB41 and *Bryobacter*, whereas the planting of ecologically restored plants significantly increased the abundance of soil RB41 and *Bryobacter*. RB41 has been found to play an important role in regulating plant health and assisting plants in coping with adverse environmental stress (Gao et al., 2024; Li et al., 2022b). *Bryobacter* is an important beneficial bacterium for plants that plays a crucial role in regulating and promoting plant growth (Contreras et al., 2023; Li X. et al., 2022; Yang et al., 2022). This study confirms for the first time that ecologically restored plants can respond to the environmental stress caused by abandoned lead–zinc mining by enriching beneficial bacterial communities and increasing their environmental adaptability and remediation potential. In addition, mining activities have increased the abundance of *Sphingomonas*, whereas the planting of ecologically restored plants has reduced the abundance of *Sphingomonas*. *Sphingomonas* has also been found to have potential for environmental remediation and the promotion of plant growth (Asaf et al., 2020). These results indicate that plants adapt better to the environment by selectively selecting “matching” bacterial populations through nonrandom selection (Li et al., 2025). RB41 and *Thiobacillus* were significantly enriched in the rhizosphere soil of plants in mining areas compared with the same type of plant rhizosphere soil from nonmining areas. *Thiobacillus* has been found to have sulfur oxidation activity and is enriched in the rhizosphere soil of various plants (Dai et al., 2024; Osman et al., 2021). These results indicate that RB41 and *Thiobacillus* are adaptable to ecologically restored plants and have important ecological value in the process of plant ecological restoration. Research has shown that different plants also randomly enrich different microbial communities in the process of ecological restoration, including *Blastocatella*, *Pseudonocardia*, and *Dongia*. *Blastocatella* has been found to have strong heavy metal tolerance (Guo et al., 2017; Li et al., 2021), *Pseudonocardia* has extensive antibacterial and fungal activity (Riahi et al., 2022), and *Dongia* has strong environmental adaptability and has been detected in various environments (Jiang et al., 2024; Lu et al., 2022). This study is the first to analyze how different plants adapt to the environment of lead–zinc mining by assembling rhizosphere bacterial communities in both common and specific ways. In the subsequent ecological restoration process of lead–zinc mines, we can specifically screen bacterial resources that are suitable for plants for plant bacterial joint ecological restoration.

Beta diversity analysis revealed that mining activities had a significant effect on the soil community structure, leading to significant differences. After plants were subjected to ecological restoration, the soil bacterial community structure recovered to a certain degree and was similar to the community structure of the control sample. PCA based on the prediction of bacterial community function also revealed the same phenomenon; that is, mining activities significantly affect the ecological function of bacterial communities, and the planting of ecologically restored plants has a positive effect on the restoration of community function, which is consistent with previous research results (Deng et al., 2024). In addition, we also found that different plants adapt to ecological restoration environments by enriching different functions, including iron complex transport system-permitting proteins and transcription pair coupling factors. The iron complex transport system-permitting protein is related to ion transport (Raymond et al., 2015), and the transcription pair coupling factor plays an important role in bacterial transcriptional regulation (Mistry et al., 2023). This study reveals for the first time that ecologically restored plants adapt to the environment and successfully complete ecological restoration through nonrandom community assembly and functional changes. These research results provide a reference for screening ecological restoration bacterial resources and developing plant bacterial joint ecological restoration strategies.

## Data Availability

The datasets presented in this study can be found in online repositories. The names of the repository/repositories and accession number(s) can be found in the article/Supplementary material.

## References

[ref1] Asaf S. Numan M. Khan A. L. Al-Harrasi A. (2020). Sphingomonas: from diversity and genomics to functional role in environmental remediation and plant growth. Crit. Rev. Biotechnol. 40, 138–152. doi: 10.1080/07388551.2019.1709793, PMID: 31906737

[ref2] Aßhauer K. P. Wemheuer B. Daniel R. Meinicke P. (2015). Tax4Fun: predicting functional profiles from metagenomic 16S rRNA data. Bioinformatics 31, 2882–2884. doi: 10.1093/bioinformatics/btv287, PMID: 25957349 PMC4547618

[ref3] Banerjee S. van der Heijden M. G. A. (2023). Soil microbiomes and one health. Nat. Rev. Microbiol. 21, 6–20. doi: 10.1038/s41579-022-00779-w 35999468

[ref4] Bao Z. J. Wang X. M. Wang Q. F. Zou L. Peng L. X. Li L. J.. (2023). A novel method of domestication combined with ARTP to improve the reduction ability of Bacillus velezensis to Cr(VI). J. Environ. Chem. Eng. 11:109091. doi: 10.1016/j.jece.2022.109091

[ref5] Bennis M. Perez-Tapia V. Alami S. Bouhnik O. Lamin H. Abdelmoumen H.. (2022). Characterization of plant growth-promoting bacteria isolated from the rhizosphere of Robinia pseudoacacia growing in metal-contaminated mine tailings in eastern Morocco. J. Environ. Manag. 304:114321. doi: 10.1016/j.jenvman.2021.114321, PMID: 35021593

[ref6] Berg G. Köberl M. Rybakova D. Müller H. Grosch R. Smalla K. (2017). Plant microbial diversity is suggested as the key to future biocontrol and health trends. FEMS Microbiol. Ecol. 93:fix050. doi: 10.1093/femsec/fix050, PMID: 28430944

[ref7] Cai B. Chen Y. Du L. Liu Z. He L. (2021). Spent mushroom compost and calcium carbonate modification enhances phytoremediation potential of Macleaya cordata to lead-zinc mine tailings. J. Environ. Manag. 294:113029. doi: 10.1016/j.jenvman.2021.113029, PMID: 34126537

[ref8] Cao J. Guo Z. Lv Y. Xu M. Huang C. Liang H. (2023). Pollution risk prediction for cadmium in soil from an abandoned mine based on random Forest model. Int. J. Environ. Res. Public Health 20:5097. doi: 10.3390/ijerph20065097, PMID: 36982005 PMC10049454

[ref9] Chen J. Guo J. Li Z. Liang X. You Y. Li M.. (2022). Effects of an arbuscular mycorrhizal fungus on the growth of and cadmium uptake in maize grown on polluted wasteland, farmland and Slopeland soils in a Lead-zinc mining area. Toxics 10:359. doi: 10.3390/toxics10070359, PMID: 35878264 PMC9322003

[ref10] Chen T. Wen X. C. Zhang L. J. Tu S. C. Zhang J. H. Sun R. N.. (2022). The geochemical and mineralogical controls on the release characteristics of potentially toxic elements from lead/zinc (Pb/Zn) mine tailings. Environ. Pollut. 315:120328. doi: 10.1016/j.envpol.2022.120328, PMID: 36202267

[ref11] Chen T. Wen X. Zhou J. Lu Z. Li X. Yan B. (2023). A critical review on the migration and transformation processes of heavy metal contamination in lead-zinc tailings of China. Environ. Pollut. 338:122667. doi: 10.1016/j.envpol.2023.122667, PMID: 37783414

[ref12] Cheng Y. T. Zhang L. He S. Y. (2019). Plant-microbe interactions facing environmental challenge. Cell Host Microbe 26, 183–192. doi: 10.1016/j.chom.2019.07.009, PMID: 31415751 PMC6697056

[ref13] Choudhari R. Sathwara N. G. Shivgotra V. K. Patel S. Rathod R. A. Shaikh S.. (2010). Study of lead exposure to children residing near a lead-zinc mine. Indian J. Occup. Environ. Med. 14, 58–62. doi: 10.4103/0019-5278.72243, PMID: 21120083 PMC2992867

[ref14] Contreras M. J. Leal K. Bruna P. Nuñez-Montero K. Goméz-Espinoza O. Santos A.. (2023). Commonalities between the Atacama Desert and Antarctica rhizosphere microbial communities. Front. Microbiol. 14:1197399. doi: 10.3389/fmicb.2023.1197399, PMID: 37538842 PMC10395097

[ref15] Dai C. Zhang G. Lin W. Luo J. (2024). Thiobacillus sedimenti sp. nov., a chemolithoautotrophic Sulphur-oxidizing bacterium isolated from freshwater sediment. Antonie Van Leeuwenhoek 118:9. doi: 10.1007/s10482-024-02026-z, PMID: 39316198

[ref16] Dang C. C. Xie G. J. Liu B. F. Xing D. F. Ding J. Ren N. Q. (2021). Heavy metal reduction coupled to methane oxidation:mechanisms, recent advances and future perspectives. J. Hazard. Mater. 405:124076. doi: 10.1016/j.jhazmat.2020.124076, PMID: 33268204

[ref17] Deng Y. Xiao W. Xiong Z. Sha A. Luo Y. Chen X.. (2024). Assembly mechanism of rhizosphere Fungi in plant restoration in Lead zinc mining areas. Genes 15:1398. doi: 10.3390/genes15111398, PMID: 39596598 PMC11593579

[ref18] Diallo A. Hasnaoui S. E. Dallahi Y. Smouni A. Fahr M. (2024). Native plant species growing on the abandoned Zaida lead/zinc mine site in Morocco: phytoremediation potential for biomonitoring perspective. PLoS One 19:e0305053. doi: 10.1371/journal.pone.0305053, PMID: 38924033 PMC11207124

[ref19] Doku E. T. Sylverken A. A. Belford J. D. E. (2024). Rhizosphere microbiome of plants used in phytoremediation of mine tailing dams. Int. J. Phytoremediation 26, 1212–1220. doi: 10.1080/15226514.2024.2301994, PMID: 38214673

[ref20] Duan R. Lin Y. Zhang J. Huang M. Du Y. Yang L.. (2021). Changes in diversity and composition of rhizosphere bacterial community during natural restoration stages in antimony mine. PeerJ 9:e12302. doi: 10.7717/peerj.12302, PMID: 34721985 PMC8520691

[ref21] Duan Y. Zhang Y. Zhao B. (2022). Lead, zinc tolerance mechanism and phytoremediation potential of Alcea rosea (Linn.) Cavan. And Hydrangea macrophylla (Thunb.) Ser. And ethylenediaminetetraacetic acid effect. Environ. Sci. Pollut. Res. Int. 29, 41329–41343. doi: 10.1007/s11356-021-18243-2, PMID: 35088277

[ref22] Edgar R. C. (2004). MUSCLE: multiple sequence alignment with high accuracy and high throughput. Nucleic Acids Res. 32, 1792–1797. doi: 10.1093/nar/gkh340, PMID: 15034147 PMC390337

[ref23] Edgar R. C. (2013). UPARSE: highly accurate OTU sequences from microbial amplicon reads. Nat. Methods 10, 996–998. doi: 10.1038/nmeth.2604 23955772

[ref24] Fernández-Martínez R. Corrochano N. Álvarez-Quintana J. Ordóñez A. Álvarez R. Rucandio I. (2024). Assessment of the ecological risk and mobility of arsenic and heavy metals in soils and mine tailings from the Carmina mine site (Asturias, NW Spain). Environ. Geochem. Health 46:90. doi: 10.1007/s10653-023-01848-6, PMID: 38367139 PMC10874346

[ref25] Gao W. Chen X. He J. Sha A. Ren Y. Wu P.. (2024). The impact of kaolin mining activities on bacterial diversity and community structure in the rhizosphere soil of three local plants. Front. Microbiol. 15:1424687. doi: 10.3389/fmicb.2024.1424687, PMID: 39314884 PMC11417686

[ref26] Garry M. R. Shock S. S. Salatas J. Dau J. (2018). Application of a weight of evidence approach to evaluating risks associated with subsistence caribou consumption near a lead/zinc mine. Sci. Total Environ. 619–620, 1340–1348. doi: 10.1016/j.scitotenv.2017.11.149, PMID: 29734611

[ref27] Guo H. Nasir M. Lv J. Dai Y. Gao J. (2017). Understanding the variation of microbial community in heavy metals contaminated soil using high throughput sequencing. Ecotoxicol. Environ. Saf. 144, 300–306. doi: 10.1016/j.ecoenv.2017.06.048, PMID: 28645031

[ref28] Guo Z. Yang J. Li K. Shi J. Peng Y. Sarkodie E. K.. (2023). Leaching behavior of as and Pb in Lead-zinc mining waste rock under mine drainage and rainwater. Toxics 11:943. doi: 10.3390/toxics11110943, PMID: 37999595 PMC10675770

[ref29] Hall M. Beiko R. G. (2018). 16S rRNA gene analysis with QIIME2. Methods Mol. Biol. 1849, 113–129. doi: 10.1007/978-1-4939-8728-3_8, PMID: 30298251

[ref30] Han L. Chen Y. Chen M. Wu Y. Su R. Du L.. (2020). Mushroom residue modification enhances phytoremediation potential of Paulownia fortunei to lead-zinc slag. Chemosphere 253:126774. doi: 10.1016/j.chemosphere.2020.126774, PMID: 32464764

[ref31] Hassan S. Bhadwal S. S. Khan M. Sabreena Nissa K. U. Shah R.. (2024). Revitalizing contaminated lands: a state-of-the-art review on the remediation of mine-tailings using phytoremediation and genomic approaches. Chemosphere 356:141889. doi: 10.1016/j.chemosphere.2024.141889, PMID: 38583533

[ref32] He S. X. Chen J. Y. Hu C. Y. Han R. Dai Z. H. Guan D. X.. (2022). Uptake, speciation and detoxification of antimonate and antimonite in as-hyperaccumulator Pteris Cretica L. Environ. Pollut. 308:119653. doi: 10.1016/j.envpol.2022.119653, PMID: 35724945

[ref33] He S. X. Peng Y. J. Chen J. Y. Liu C. J. Cao Y. Li W.. (2023). Antimony uptake and speciation, and associated mechanisms in two as-hyperaccumulators Pteris vittata and Pteris cretica. J. Hazard. Mater. 455:131607. doi: 10.1016/j.jhazmat.2023.131607, PMID: 37182466

[ref34] Jacob J. M. Karthik C. Saratale R. G. Kumar S. S. Prabakar D. Kadirvelu K.. (2018). Biological approaches to tackle heavy metal pollution: a survey of literature. J. Environ. Manag. 217, 56–70. doi: 10.1016/j.jenvman.2018.03.077 29597108

[ref35] Jiang S. Chen T. Zhang J. Duan L. X. Yan B. (2022). Roasted modified lead-zinc tailings using alkali as activator and its mitigation of cd contaminated: characteristics and mechanisms. Chemosphere 297:134029. doi: 10.1016/j.chemosphere.2022.134029, PMID: 35231475

[ref36] Jiang X. Guo Y. Li H. Li X. Liu J. (2022). Ecological evolution during the three-year restoration using rhizosphere soil cover method at a Lead-zinc tailing pond in karst areas. Sci. Total Environ. 853:158291. doi: 10.1016/j.scitotenv.2022.158291, PMID: 36030848

[ref37] Jiang X. Ruan L. Wu N. Mao D. He J. Wang S.. (2024). Dongia sedimenti sp. nov., isolated from river sediment. Int. J. Syst. Evol. Microbiol. 74:006532. doi: 10.1099/ijsem.0.006532 39312393

[ref38] Junusbekov M. M. Akbasova A. D. Seidakbarova A. D. Koishiyeva G. Z. Sainova G. A. (2023). Ecological assessment of soil contamination by heavy metals affected in the past by the lead-zinc mining and processing complex in Kentau, Kazakhstan. Environ. Monit. Assess. 195:586. doi: 10.1007/s10661-023-11189-7, PMID: 37074563

[ref39] Kan X. Dong Y. Feng L. Zhou M. Hou H. (2021). Contamination and health risk assessment of heavy metals in China's lead-zinc mine tailings: a meta-analysis. Chemosphere 267:128909. doi: 10.1016/j.chemosphere.2020.128909, PMID: 33187663

[ref40] Kastury F. Cahill G. Fernando A. Brotodewo A. Huang J. Juhasz A. L.. (2023). Metallic mangroves: sediments and in situ diffusive gradients in thin films (DGTs) reveal Avicennia marina (Forssk.) Vierh. Lives with high contamination near a lead-zinc smelter in South Australia. Sci. Total Environ. 857:159503. doi: 10.1016/j.scitotenv.2022.159503, PMID: 36265646

[ref41] Larsen T. S. Kristensen J. A. Asmund G. Bjerregaard P. (2001). Lead and zinc in sediments and biota from Maarmorilik, West Greenland: an assessment of the environmental impact of mining wastes on an Arctic fjord system. Environ. Pollut. 114, 275–283. doi: 10.1016/s0269-7491(00)00214-1, PMID: 11504350

[ref42] Lei C. Yan B. Chen T. Xiao X. M. (2018). Preparation and adsorption characteristics for heavy metals of active silicon adsorbent from leaching residue of lead-zinc tailings. Environ. Sci. Pollut. Res. Int. 25, 21233–21242. doi: 10.1007/s11356-018-2194-9, PMID: 29779079

[ref43] Li Y. J. Fu H. Zhang J. Y. Zhang Z. X. Li J. K. (2021). Study of pollutant accumulation characteristics and microbial community impact at three bioretention facilities. Environ. Sci. Pollut. Res. Int. 28, 44389–44407. doi: 10.1007/s11356-021-13801-0, PMID: 33847886

[ref44] Li X. Li B. Jin T. Chen H. Zhao G. Qin X.. (2022). Rhizospheric microbiomics integrated with plant transcriptomics provides insight into the cd response mechanisms of the newly identified cd accumulator Dahlia pinnata. Front. Plant Sci. 13:1091056. doi: 10.3389/fpls.2022.1091056, PMID: 36589044 PMC9798219

[ref45] Li Y. Luo J. Yu J. Xia L. Zhou C. Cai L.. (2018). Improvement of the phytoremediation efficiency of Neyraudia reynaudiana for lead-zinc mine-contaminated soil under the interactive effect of earthworms and EDTA. Sci. Rep. 8:6417. doi: 10.1038/s41598-018-24715-2, PMID: 29686313 PMC5913105

[ref46] Li D. Ramos A. O. Bah A. Li F. (2024). Valorization of lead-zinc mine tailing waste through geopolymerization: synthesis, mechanical, and microstructural properties. J. Environ. Manag. 349:119501. doi: 10.1016/j.jenvman.2023.119501, PMID: 37952378

[ref47] Li Q. Wu Q. Zhang T. Xiang P. Bao Z. J. Tu W. Y.. (2022a). Phosphate mining activities affect crop rhizosphere fungal communities. Sci. Total Environ. 838:156196. doi: 10.1016/j.scitotenv.2022.156196 35623536

[ref48] Li Q. Xiang P. Li L. Zhang T. Wu Q. Bao Z.. (2024). Phosphorus mining activities alter endophytic bacterial communities and metabolic functions of surrounding vegetables and crops. Plant Soil 497, 155–174. doi: 10.1007/s11104-023-05961-4

[ref49] Li Q. Xiang P. Zhang T. Wu Q. Bao Z. Tu W.. (2022b). The effect of phosphate mining activities on rhizosphere bacterial communities of surrounding vegetables and crops. Sci. Total Environ. 821:153479. doi: 10.1016/j.scitotenv.2022.153479, PMID: 35092784

[ref50] Li Q. Yuan W. Deng X. Chen Y. Li L. Chen L.. (2025). High lead-tolerant mutant Bacillus tropicus AT31-1 from rhizosphere soil of Pu-erh and its remediation mechanism. Bioresour. Technol. 416:131751. doi: 10.1016/j.biortech.2024.131751, PMID: 39521187

[ref51] Liu K. Zhang H. Liu Y. Li Y. Yu F. (2020). Investigation of plant species and their heavy metal accumulation in manganese mine tailings in Pingle Mn mine, China. Environ. Sci. Pollut. Res. Int. 27, 19933–19945. doi: 10.1007/s11356-020-08514-9, PMID: 32232756

[ref52] Lu C. Y. Dong L. Wang D. Li S. Fang B. Z. Han M. X.. (2022). Dongia deserti sp. nov., isolated from the Gurbantunggut Desert soil. Curr. Microbiol. 79:342. doi: 10.1007/s00284-022-03051-9 36209298

[ref53] Luo Z. Tang C. Hao Y. Wang Z. Yang G. Wang Y.. (2022). Solidification/stabilization of heavy metals and its efficiency in lead-zinc tailings using different chemical agents. Environ. Technol. 43, 1613–1623. doi: 10.1080/09593330.2020.1845817, PMID: 33135954

[ref54] Ma Y. Li C. Yan J. Yu H. Kan H. Yu W.. (2023). The release analysis of as and Cr metals in lead-zinc smelting slag: mineralogical analysis, bioavailability and leachability analysis. Environ. Res. 229:115751. doi: 10.1016/j.envres.2023.115751 36966997

[ref55] Magoc T. Salzberg S. L. (2011). FLASH: fast length adjustment of short reads to improve genome assemblies. Bioinformatics 27, 2957–2963. doi: 10.1093/bioinformatics/btr507, PMID: 21903629 PMC3198573

[ref56] Mistry H. Kumari S. Aswal V. K. Gupta G. D. (2023). Structural characterization of transcription-coupled repair protein UVSSA and its interaction with TFIIH protein. Int. J. Biol. Macromol. 247:125792. doi: 10.1016/j.ijbiomac.2023.125792, PMID: 37442507

[ref57] Narayanan M. Natarajan D. Kandasamy G. Kandasamy S. Shanmuganathan R. Pugazhendhi A. (2021). Phytoremediation competence of short-term crops on magnesite mine tailing. Chemosphere 270:128641. doi: 10.1016/j.chemosphere.2020.128641, PMID: 33121805

[ref58] Noor I. Sohail H. Sun J. Nawaz M. A. Li G. Hasanuzzaman M.. (2022). Heavy metal and metalloid toxicity in horticultural plants: tolerance mechanism and remediation strategies. Chemosphere 303:135196. doi: 10.1016/j.chemosphere.2022.135196, PMID: 35659937

[ref59] Ojuederie O. B. Babalola O. O. (2017). Microbial and plant-assisted bioremediation of heavy metal polluted environments: a review. Int. J. Environ. Res. Public Health 14:1504. doi: 10.3390/ijerph14121504, PMID: 29207531 PMC5750922

[ref60] Osman J. R. Cardon H. Montagnac G. Picard A. Daniel I. (2021). Pressure effects on sulfur-oxidizing activity of Thiobacillus thioparus. Environ. Microbiol. Rep. 13, 169–175. doi: 10.1111/1758-2229.12922, PMID: 33421329 PMC7986089

[ref61] Paluchamy B. Mishra D. P. (2022). Dust pollution hazard and harmful airborne dust exposure assessment for remote LHD operator in underground lead-zinc ore mine open stope. Environ. Sci. Pollut. Res. Int. 29, 89585–89596. doi: 10.1007/s11356-022-22059-z, PMID: 35852746

[ref62] Peng J. Y. Zhang S. Wang Y. J. Zhao R. F. Zhou Y. L. Zhou J. W. (2023). Identification of priority pollutants and key factors affecting environmental risks of lead-zinc mine tailing sites. Sci. Total Environ. 889:164039. doi: 10.1016/j.scitotenv.2023.164039, PMID: 37211123

[ref63] Qiao Y. Hou H. Chen L. Wang H. Jeyakumar P. Lu Y.. (2022). Comparison of Pb and cd in wheat grains under air-soil-wheat system near lead-zinc smelters and total suspended particulate introduced modeling attempt. Sci. Total Environ. 839:156290. doi: 10.1016/j.scitotenv.2022.156290, PMID: 35644402

[ref64] Qiu L. Sha A. Li N. Ran Y. Xiang P. Zhou L.. (2024). The characteristics of fungal responses to uranium mining activities and analysis of their tolerance to uranium. Ecotoxicol. Environ. Saf. 277:116362. doi: 10.1016/j.ecoenv.2024.116362, PMID: 38657459

[ref65] Quast C. Pruesse E. Yilmaz P. Gerken J. Schweer T. Yarza P.. (2013). The SILVA ribosomal RNA gene database project: improved data processing and web-based tools. Nucleic Acids Res. 41, D590–D596. doi: 10.1093/nar/gks1219, PMID: 23193283 PMC3531112

[ref66] Raymond K. N. Allred B. E. Sia A. K. (2015). Coordination chemistry of microbial Iron transport. Acc. Chem. Res. 48, 2496–2505. doi: 10.1021/acs.accounts.5b00301, PMID: 26332443 PMC4576731

[ref67] Riahi H. S. Heidarieh P. Fatahi-Bafghi M. (2022). Genus Pseudonocardia: what we know about its biological properties, abilities and current application in biotechnology. J. Appl. Microbiol. 132, 890–906. doi: 10.1111/jam.15271 34469043

[ref68] Sha H. Li J. Wang L. Nong H. Wang G. Zeng T. (2023). Preparation of phosphorus-modified biochar for the immobilization of heavy metals in typical lead-zinc contaminated mining soil: performance, mechanism and microbial community. Environ. Res. 218:114769. doi: 10.1016/j.envres.2022.114769, PMID: 36463989

[ref69] Singh A. Chauhan S. Varjani S. Pandey A. Bhargava P. C. (2022). Integrated approaches to mitigate threats from emerging potentially toxic elements: a way forward for sustainable environmental management. Environ. Res. 209:112844. doi: 10.1016/j.envres.2022.112844, PMID: 35101398

[ref70] Su R. Ou Q. Wang H. Dai X. Chen Y. Luo Y.. (2023). Organic-inorganic composite modifiers enhance restoration potential of Nerium oleander L. to lead-zinc tailing: application of phytoremediation. Environ. Sci. Pollut. Res. Int. 30, 56569–56579. doi: 10.1007/s11356-023-26359-w, PMID: 36920611

[ref71] Su R. Ou Q. Wang H. Luo Y. Dai X. Wang Y.. (2022). Comparison of phytoremediation potential of Nerium indicum with inorganic modifier calcium carbonate and organic modifier mushroom residue to Lead-zinc tailings. Int. J. Environ. Res. Public Health 19:10353. doi: 10.3390/ijerph191610353, PMID: 36011987 PMC9408432

[ref72] Sun R. Gao Y. Yang Y. (2022). Leaching of heavy metals from lead-zinc mine tailings and the subsequent migration and transformation characteristics in paddy soil. Chemosphere 291:132792. doi: 10.1016/j.chemosphere.2021.132792, PMID: 34748803

[ref73] Tang C. Chen Y. Zhang Q. Li J. Zhang F. Liu Z. (2019). Effects of peat on plant growth and lead and zinc phytostabilization from lead-zinc mine tailing in southern China: screening plant species resisting and accumulating metals. Ecotoxicol. Environ. Saf. 176, 42–49. doi: 10.1016/j.ecoenv.2019.03.078, PMID: 30921695

[ref74] Tang B. Xu H. Song F. Ge H. Yue S. (2022). Effects of heavy metals on microorganisms and enzymes in soils of lead-zinc tailing ponds. Environ. Res. 207:112174. doi: 10.1016/j.envres.2021.112174, PMID: 34637758

[ref75] Thomas G. Sheridan C. Holm P. E. (2022). A critical review of phytoremediation for acid mine drainage-impacted environments. Sci. Total Environ. 811:152230. doi: 10.1016/j.scitotenv.2021.152230, PMID: 34896134

[ref76] Trivedi P. Leach J. E. Tringe S. G. Sa T. Singh B. K. (2020). Plant-microbiome interactions: from community assembly to plant health. Nat. Rev. Microbiol. 18, 607–621. doi: 10.1038/s41579-020-0412-1 32788714

[ref77] Wang H. Ju C. Zhou M. Chen J. Kan X. Dong Y.. (2022). Acid rain-dependent detailed leaching characteristics and simultaneous immobilization of Pb, Zn, Cr, and cd from hazardous lead-zinc tailing. Environ. Pollut. 307:119529. doi: 10.1016/j.envpol.2022.119529 35623574

[ref78] Wang Q. Wang H. Ma Y. Wang J. Su W. Xiao E.. (2023). Geochemical distributions of natural radionuclides in surface soils and sediments impacted by lead-zinc mining activity. Ecotoxicol. Environ. Saf. 263:115210. doi: 10.1016/j.ecoenv.2023.115210, PMID: 37418943

[ref79] Xiao Y. Chen L. Li C. Ma J. Chen R. Yang B.. (2022). Role of the rhizosphere bacterial community in assisting phytoremediation in a lead-zinc area. Front. Plant Sci. 13:1106985. doi: 10.3389/fpls.2022.1106985, PMID: 36874912 PMC9982732

[ref80] Xiao W. Zhang Y. Chen X. Sha A. Xiong Z. Luo Y.. (2024). The diversity and community composition of three plants' rhizosphere fungi in Kaolin mining areas. J. Fungi (Basel) 10:306. doi: 10.3390/jof10050306 38786661 PMC11121986

[ref81] Xiong Z. Zhang Y. Chen X. Sha A. Xiao W. Luo Y.. (2024). Impact of vanadium-titanium-magnetite mining activities on endophytic bacterial communities and functions in the root Systems of Local Plants. Genes (Basel) 15:526. doi: 10.3390/genes15050526, PMID: 38790155 PMC11121153

[ref82] Xu Q. Liu Z. Chen Y. Qin L. Zhao M. Tang W.. (2024). Serum metabolic changes link metal mixture exposures to vascular endothelial inflammation in residents living surrounding rivers near abandoned lead-zinc mines. Environ. Pollut. 358:124493. doi: 10.1016/j.envpol.2024.124493, PMID: 38960116

[ref83] Yang L. Ge S. Liu J. Iqbal Y. Jiang Y. Sun R.. (2023). Spatial distribution and risk assessment of heavy metal(oid)s contamination in topsoil around a Lead and zinc smelter in Henan Province, Central China. Toxics 11:427. doi: 10.3390/toxics11050427, PMID: 37235242 PMC10224199

[ref84] Yang Z. N. Liu Z. S. Wang K. H. Liang Z. L. Abdugheni R. Huang Y.. (2022). Soil microbiomes divergently respond to heavy metals and polycyclic aromatic hydrocarbons in contaminated industrial sites. Environ. Sci. Ecotechnol. 10:100169. doi: 10.1016/j.ese.2022.100169, PMID: 36159729 PMC9488039

[ref85] Yohannes Y. B. Nakayama S. M. M. Yabe J. Toyomaki H. Kataba A. Nakata H.. (2022). Glutathione S-transferase gene polymorphisms in association with susceptibility to lead toxicity in lead- and cadmium-exposed children near an abandoned lead-zinc mining area in Kabwe, Zambia. Environ. Sci. Pollut. Res. Int. 29, 6622–6632. doi: 10.1007/s11356-021-16098-1, PMID: 34453679

